# Design and Synthesis of Fe_3_O_4_-Loaded Polymer Microspheres with Controlled Morphology: Section II Fabrication of Walnut-like Superparamagnetic Polymer Microspheres

**DOI:** 10.3390/polym17131876

**Published:** 2025-07-05

**Authors:** Florence Acha, Talya Scheff, Nathalia DiazArmas, Jinde Zhang

**Affiliations:** Department of Plastics Engineering, University of Massachusetts Lowell, Lowell, MA 01854, USA; florence_acha@student.uml.edu (F.A.); talya_scheff@student.uml.edu (T.S.); nathalia_diazarmas@student.uml.edu (N.D.)

**Keywords:** walnut-like polymer microspheres, mini-emulsion polymerization, superparamagnetic, Fe_3_O_4_ nanoparticles

## Abstract

A simple and innovative synthesis strategy was established to produce polymer microspheres with a distinctive walnut-like morphology, incorporating Fe_3_O_4_ nanoparticles within their structure. This was achieved through γ-ray-initiated mini-emulsion polymerization. To ensure high encapsulation efficiency, the surface of the Fe_3_O_4_ nanoparticles was chemically altered to shift their wettability from hydrophilic to hydrophobic, enabling uniform dispersion within the monomer phase before polymerization. The formation of the walnut-like architecture was found to be significantly influenced by both the polymerization dynamics and phase separation, as well as the shrinkage of the crosslinked polymer network formed between the monomer and the resulting polymer. Divinylbenzene (DVB) was chosen as the monomer due to its ability to generate a mechanically stable polymer framework. The γ-ray irradiation effectively initiated polymerization while preserving structural coherence. A detailed analysis using FTIR, SEM, and TEM confirmed the successful fabrication of the Fe_3_O_4_-loaded polymer microspheres with their characteristic textured surface. Moreover, magnetic characterization via vibrating sample magnetometry (VSM) indicated pronounced superparamagnetic behavior and strong magnetic responsiveness, highlighting the potential of these microspheres for advanced biomedical applications.

## 1. Introduction

Magnetic polymer composite particles (MPCPs) have garnered widespread attention due to their unique combination of magnetic functionality and polymeric particle structures. This hybrid nature imparts a wide range of capabilities, making them promising candidates for use in areas such as biomedical engineering, bioengineering, magnetic hyperthermia, MRI diagnostics, and enzyme immobilization [1]. Common methods for producing MPCPs generally fall into three categories: physical encapsulation, the in situ formation of magnetic components, and the polymerization of monomers in the presence of magnetic materials.

A considerable amount of research has been dedicated to manipulating the shape and structure of micro- and nanoscale particles using a range of emulsion-based methods and polymerization techniques [2]. More recently, there has been growing interest in polymer microspheres exhibiting well-defined and unusual morphologies, driven by their potential in diverse applications such as catalysis [3], photonic crystal engineering [4,5], self-assembled systems [6], sensing technologies [7], and molecular recognition processes [7]. Despite this progress, the reliable synthesis of such intricately structured microspheres continues to present substantial challenges.

From a thermodynamic perspective, smooth-surfaced spherical particles are energetically favorable and thus form more readily. As a result, the development of non-spherical particles has often depended on reshaping or modifying existing spherical templates. Techniques such as seeded polymerization and the self-assembly of complex structures have been employed to produce a variety of particle morphologies—including ellipsoids [8], hollow forms [9], snowman-like geometries [10,11], and disks [12]. Colloid-based and interfacial lithographic methods [12] have also been investigated for this purpose. Despite these advances, most reported particles still feature smooth surfaces, and generating non-spherical particles with textured or rough surfaces—which are desirable due to their higher surface area—remains a considerable challenge.

A significant advancement in this area involved the creation of polymer particles with a walnut-like, multi-hollow architecture [13]. This was accomplished using sulfonated and crosslinked polystyrene spheres as sacrificial templates, prepared via dispersion polymerization followed by sulfonation [13]. Nonetheless, the multistep nature of this fabrication route introduces complexity and limits reproducibility.

Magnetic polymer microspheres have emerged as versatile materials for applications in a broad range of fields, including biomedical engineering [14,15], magnetic hyperthermia [16], MRI diagnostics [17], enzyme immobilization [18], catalysis [19], cellular and genetic separation [20], and environmental cleanup [21]. Combining magnetic functionality with engineered surface textures could unlock additional capabilities in these domains.

In previous work [22], we successfully fabricated magnetic polymer composite particles (MPCPs) with encapsulated Fe_3_O_4_ via γ-ray induced mini-emulsion polymerization and found that the hydrophobic modification of Fe_3_O_4_ enabled high encapsulation, while optimal SDS (surfactant) and CA (co-stabilizer) levels ensured narrow size distribution. In that work, we focused on MPCPs with a smooth sphere shape. This work is the continuation of our previous work but focused on the fabrication of MPCPs with rough surfaces—especially those with a walnut-like shape—by leveraging the knowledge we obtained from previous work. The procedure and formulation to fabricate walnut-like poly (divinyl benzene)/Fe_3_O_4_ microspheres were modified from our previous work to make MPCPs [22]. Differing from previous work using cetyl alcohol (CA), here we used hexadecane (HD) as a co-stabilizer to make our mini-emulsion system. This is because HD showed better solubility in our monomer DVB. We also manipulated the irradiation time to control the surface roughness of MPCPS.

## 2. Materials and Methods

### 2.1. Materials

Divinyl benzene (DVB) and styrene were purified by passing them through an alumina column to eliminate any residual radical inhibitors. Following purification, the monomers were stored at approximately 4 °C in a refrigerator until needed. All other reagents used in this study—including ferrous sulfate heptahydrate (FeSO_4_·7H_2_O), ferric chloride (FeCl_3_), 25 wt.% aqueous ammonia, oleic acid, hexadecane (HD), sodium dodecyl sulfate (SDS), and ethanol—were of analytical grade and utilized as received, without any additional purification steps.

### 2.2. MNPs Synthesis and Modification

Magnetic nanoparticles (MNPs) synthesis and modification were performed following the method presented in our previous work [22]. Initially, 8.35 g of FeSO_4_·7H_2_O and 7.31 g of FeCl_3_ were dissolved in 30 mL of distilled water to form a uniform solution, which was then diluted with an additional 70 mL of distilled water. The resulting mixture was heated to 85 °C under continuous stirring. To initiate precipitation, 30 mL of 25 wt.% aqueous ammonia was added. After one minute, 2 g of oleic acid was slowly introduced to modify the nanoparticle surfaces. The reaction was maintained for three hours. The resulting black solid was collected using magnetic separation and washed sequentially with water and ethanol. This purification step was repeated three more times via magnetic decantation to ensure the removal of residual impurities.

### 2.3. Preparation of Walnut-like Poly (Divinyl Benzene)/Fe_3_O_4_ Microspheres

The procedure and formulation used to fabricate walnut-like poly (divinyl benzene)/Fe_3_O_4_ microspheres was modified from our previous work to make MPCPs [22]. Differing from previous work using cetyl alcohol (CA), here we used hexadecane (HD) as the co-stabilizer to make our mini-emulsion system. This is because HD shows better solubility in our monomer DVB. In a standard procedure, 0.10 g of magnetic nanoparticles (MNPs) and 0.4 g of hexadecane (HD) were measured and dispersed into 3 g of divinylbenzene (DVB) monomer via ultrasonication. Separately, 0.06 g of sodium dodecyl sulfate (SDS) was dissolved in 30 mL of distilled water. Once both solutions were homogeneously mixed, the emulsification process was initiated by gradually adding the oil phase (DVB + HD + MNPs) into the aqueous phase (water + SDS) while stirring vigorously at 1000 rpm for one hour. Following emulsification, mini-emulsification was conducted by subjecting the prepared emulsion to ultrasonic treatment in a water bath for 10 min. To prevent premature polymerization, ice was introduced to keep the temperature low during this process. Prior to initiating polymerization, nitrogen gas was bubbled through the mini-emulsion to remove any dissolved oxygen, after which the system was sealed in a 100 mL glass bottle with a narrow neck. Polymerization was then carried out utilizing γ-ray irradiation from a ^60^Co source at a specific dose rate of 50 Gy/min at room temperature (23 °C). Total absorbed dose was varied for each sample by controlling the irradiation time to study its effect on the final morphology of the prepared MPCPs. After irradiation, the fabricated MPCPs were separated by placing the solution on a magnet for 20 min. The upper liquid was carefully removed and preserved for characterization as a reference. The MPCPs were then washed multiple times with distilled water and ethanol using magnetic decantation, followed by air-drying at room temperature.

To determine the mechanism of formation of walnut-like particles, a series of experiments were performed, as shown in Table 1.

### 2.4. Characterization

In this work, various analytical techniques were employed to characterize the composition of the walnut-like polymer microspheres. Fourier transform infrared (FTIR) spectroscopy was used to identify functional groups present in MPCPs. Spectra were obtained using a Bruker Vector-22 instrument (Bruker Corporation, Billerica, MA, USA), with samples prepared by the KBr pellet method.

Thermogravimetric analysis (TGA) was utilized to quantify the MNPs loading level in MPCPs. These measurements were carried out using a Shimadzu TGA-50H system (Shimadzu Corporation, Kyoto, Japan) under an air atmosphere, with a temperature ramp from 50 °C to 700 °C at a rate of 10 °C/min, during which weight loss was continuously monitored.

The internal structure and spatial distribution of MNPs within the polymer matrix were visualized through transmission electron microscopy (TEM) using a Hitachi H-7650 (Hitachi High-Technologies Corporation, Tokyo, Japan), operating at an accelerating voltage of 200 kV on ultrathin (~70 nm) microtomed sections.

The surface morphology and fine structural features of the microspheres were further examined using field-emission scanning electron microscopy (FESEM, JEOL JSM-6700F, JEOL Ltd, Tokyo, Japan) at 10 kV. Finally, the magnetic properties of both the isolated nanoparticles and the resulting hybrid microspheres were evaluated at room temperature using a magnetic property measurement system (MPMS XL, Quantum Design Corp., San Diego, California, US), with the applied magnetic field swept in the sequence 0 → +1 T → 0 → −1 T → 0.

## 3. Results and Discussion

Since the oleic acid-modified magnetic nanoparticles (MNPs) used in this work were the same as those used in our previous work [22], we suggest referring to [22] for all the characterizations related to them. In this work, we will only focus on the results related to our fabricated MPCPs.

**Figure 1:**
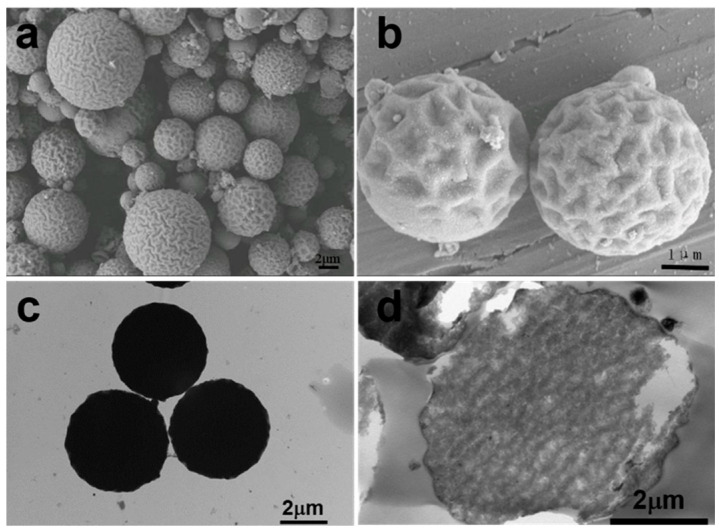
(**a**,**b**) SEM images; (**c**) TEM images of cross-section; (**d**) TEM image of walnut-like magnetic polymer microspheres (S-4).

As shown in Figure 1, microspheres with a distinct rough surface were successfully synthesized through γ-ray irradiation-induced mini-emulsion polymerization. Due to their corrugated and folded surface, resembling the texture of a walnut, this morphology is referred to as “walnut-like” in this study. The resulting microspheres exhibit a size distribution ranging from 2 to 6 μm. Since no agitation was applied during irradiation, the stability of the mini-emulsion was affected during polymerization, leading to a variation in microsphere sizes, which influenced their overall morphology.

**Figure 2:**
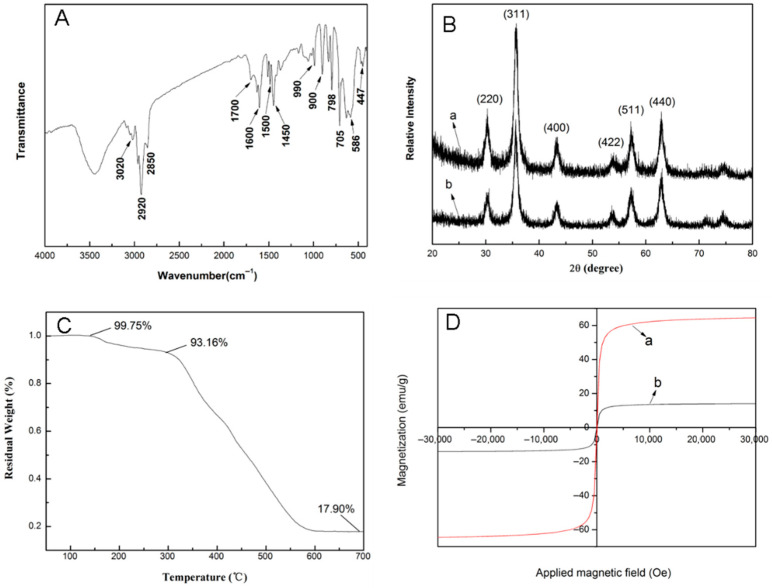
FTIR spectrum of S-4 in Table 1: (**A**) FTIR spectrum of S-4; (**B**) XRD of (a) S-4 and (b) MNPs; (**C**) TGA curve; and (**D**) Hysteresis loop measurement of (a) MNPs and (b) walnut-like magnetic polymer microspheres.

Beyond morphological evaluation, a range of analytical techniques was employed to thoroughly characterize sample S-4. The FTIR spectrum (Figure 2A, adapted from Scheff et al. [22]) confirms the presence of poly(vinyl-divinylbenzene) (PVDB). Characteristic absorption bands at 705 and 798 cm^−1^ are linked to the out-of-plane bending of aromatic C–H bonds, while the bands near 1450, 1500, and 1600 cm^−1^ are assigned to aromatic C–C stretching vibrations. The signal at 3020 cm^−1^ corresponds to C–H stretching in the benzene ring. Peaks located at 2850 and 2920 cm^−1^ are attributed to the stretching of saturated C–H bonds, originating either from –CH_2_ groups in the polymer matrix or –R groups from oleic acid. Absorptions at 990 and 900 cm^−1^ are due to C–O–C stretching, indicative of oleic acid residues. Additionally, the bands at 586 and 447 cm^−1^ are consistent with Fe^2+^–O^2−^ and Fe^3+^–O^2−^ interactions, reflecting the presence of magnetic nanoparticles.

To gain further insight into the structural properties of the magnetic phase, X-ray diffraction (XRD) analysis was carried out on both the bare MNPs and the composite S-4 (Figure 2B). The diffraction peaks observed for the unmodified MNPs (Figure 2B(b)) match the standard pattern of magnetite (Fe_3_O_4_), as documented in JCPDS card no. 74-0748. These same peaks appear in the S-4 spectrum, suggesting that the crystalline structure of the MNPs remains intact during synthesis. A slight increase in the background signal at low 2θ angles in S-4 suggests the inclusion of amorphous PVDB.

Thermogravimetric analysis (Figure 2C) provided further information on the composition of S-4. The initial weight loss of 6.84% below 300 °C is attributed to the evaporation of water, surfactants, co-stabilizers, and loosely bound oleic acid. Between 300 °C and 600 °C, a major mass reduction of 75.26% occurs, corresponding to the thermal decomposition of PVDB and strongly bonded oleic acid on MNPs (see Figure S2). The remaining 17.90% is attributed to the inorganic magnetic core. Based on these values, the actual content of bared MNPs in S-4 is calculated to be approximately 19.21%, derived from the ratio of residual weight to initial organic mass.

Magnetic measurements were performed on both the pure MNPs and the S-4 microspheres at room temperature (300 K), as shown in Figure 2D. Both materials exhibited zero remnant magnetization, indicating superparamagnetic behavior—a property that minimizes aggregation in the absence of an external magnetic field. The saturation magnetization values were found to be roughly 60 emu/g (Figure 2D, top red line) for the bare MNPs and 12 emu/g for S-4 (Figure 2D(b) bottom black line). Based on these values, the estimated MNP content in the composite is approximately 20 wt.%, closely matching the TGA-based estimate and affirming the effective embedding of superparamagnetic particles within the polymer matrix. This high magnetic loading enhances the composite’s potential utility in diverse fields, such as biomedical engineering, environmental technology, and pharmaceuticals.

### 3.1. Formation Mechanism of Walnut-like Superparamagnetic Microspheres

To investigate the formation mechanism of the walnut-like morphology, we monitored the polymerization reaction by varying the net absorbed dose. Additionally, we adjusted certain components and their concentrations to assess their influence on the final product. Details of these changes are provided in Table 1. The results indicated that several factors, including the absorbed dose and monomer type, significantly impacted the final morphology of the microspheres, as discussed in the following paragraphs.

**Figure 3:**
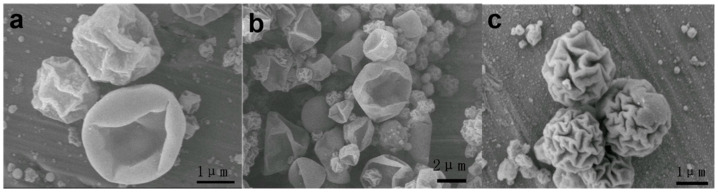
SEM images of magnetic polymer particles at different radiation times: (**a**) S-1 (6 h), (**b**) S-2 (16 h), and (**c**) S-3 (22 h).

**Figure 4:**
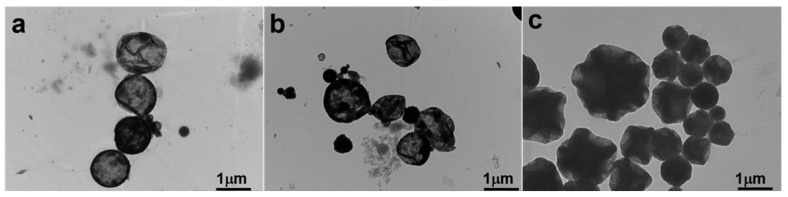
TEM images of magnetic polymer particles at different radiation times: (**a**) S-1 (6 h), (**b**) S-2 (16 h), and (**c**) S-3 (22 h).

In this study, the degree of polymerization of DVB was directly related to the absorbed dose, which, in turn, was proportional to the irradiation time. We hypothesized that higher doses would result in a higher degree of polymerization. As shown in Figure 3, walnut-like microspheres began to form after 22 h of irradiation (~66 kGy absorbed dose). When the irradiation time did not exceed 16 h, hollow microspheres with smooth surfaces were observed (Figure 3a,b). At lower absorbed doses, the polymerization degree of the monomers was limited due to the shorter irradiation times and fewer active radicals being generated. This resulted in more DVB monomers remaining inside the microspheres, with thinner PDVB walls. These hollow microspheres, having thinner walls (Figure 4a,b), were more likely to collapse during subsequent washing and drying steps. Conversely, longer irradiation times led to a higher degree of polymerization and a lower amount of unreacted monomer, which resulted in solid microspheres (Figure 3c and Figure 4c). This prevented the collapse of PDVB microspheres. The unique walnut-like roughness observed on the microsphere surfaces (Figure 1a,b and Figure 4c) can be attributed to late-stage polymerization, where DVB monomers were excluded from the crosslinked PDVB network. This exclusion caused the network to shrink, forming a corrugated surface.

**Figure 5:**
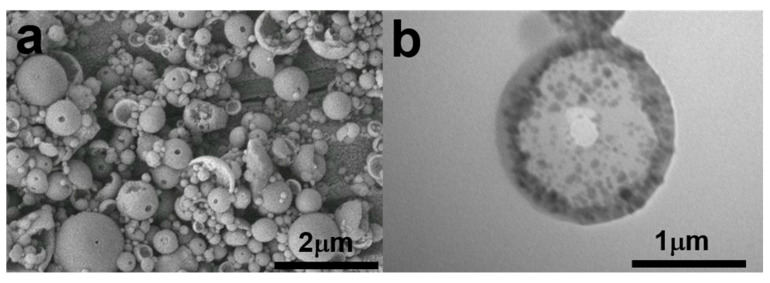
(**a**) Scanning electron microscopy (SEM) image; (**b**) transmission electron microscopy (TEM) image of sample S-5, as defined in Table 1.

To further validate this, we replaced DVB with styrene and conducted the same experiment as for S-4 (Table 1). The results in Figure 5 show that S-5, made solely from styrene monomer, produced hollow microspheres with a single hole and smooth surfaces. No walnut-like morphology was observed. In contrast, S-4, prepared with a DVB monomer, exhibited a distinct walnut-like surface (Figure 1a,b) and a condensed internal structure, as shown by the TEM image of the cross-section of the walnut-like microspheres (Figure 1d). This difference can be attributed to the better solubility of PS in styrene compared to PDVB in DVB, which prevents phase separation during polymerization. In contrast, phase separation and the contraction of the crosslinked network between PDVB and DVB, combined with the low degree of hollowing due to the high degree of polymerization, led to the formation of a walnut-like morphology.

#### Hypothesis for Walnut-like MPCPs Formation

**Figure 6:**
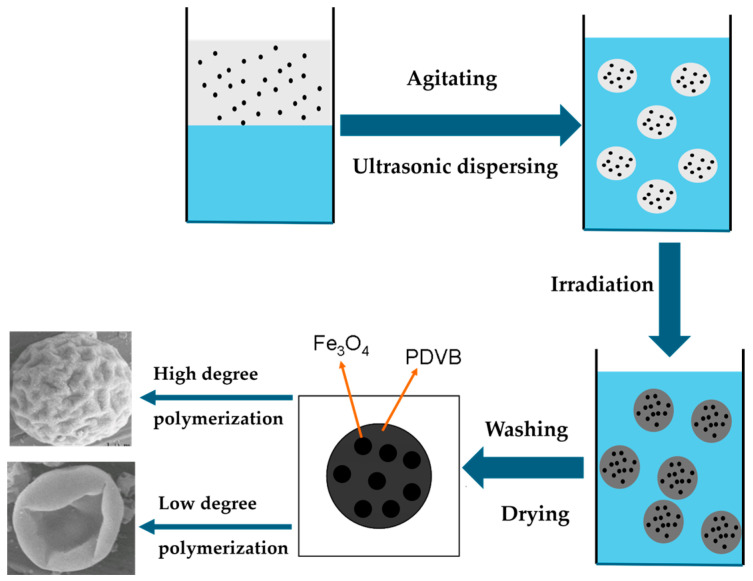
Illustration of the formation mechanism of walnut-like MPCPs.

Based on our experimental results, we proposed a hypothesis to explain the formation of walnut-like MPCPs and created an illustration of it, as shown in Figure 6. In our study, a mini-emulsion served as the soft template, and γ-radiation was used to initiate the polymerization and crosslinking of DVB. Therefore, the reaction likely begins at the oil–water interface and propagates inward toward the center of the oil droplets. At shorter irradiation times (e.g., S-1 and S-2), a larger amount of unreacted DVB remains, resulting in the formation of hollow MPCPs with thin PDVB shells. These thin-walled structures are prone to collapse during the subsequent washing and drying processes. With moderate irradiation (e.g., S-3), the extent of polymerization increases, leading to thicker shells. At this stage, the walnut-like surface morphology begins to emerge, though the particles may still collapse due to their hollow interiors. Upon further increases in the irradiation time, the polymerization reaction reaches completion, leaving few unreacted monomers and yielding solid MPCPs with a walnut-like morphology. When we replaced DVB with styrene, neither collapsed spheres nor solid spheres with walnut-like morphology were observed. Instead, we found broken hollow microspheres or intact, smooth spheres with a hole. This difference can be attributed to the higher solubility of polystyrene (PS) in styrene compared to poly(divinylbenzene) (PDVB) in DVB, which inhibits phase separation during polymerization. In contrast, the formation of the walnut-like morphology in the DVB system likely results from a combination of phase separation, contraction of the crosslinked PDVB network, and a high degree of polymerization that limits hollowing. Therefore, the walnut-like morphology is probably driven by the interplay of high polymerization degree, phase separation, and network contraction [23]. Further experiments are needed to fully validate this hypothesis. These could include increasing the polymerization time in the styrene system, partially substituting DVB with styrene, or initiating polymerization within the monomer droplet using an internal initiator such as AIBN (Azobisisobutyronitrile). These directions will be explored in future work.

## 4. Conclusions

This study investigates radiation-induced mini-emulsion polymerization for synthesizing walnut-like PDVB/Fe_3_O_4_ microspheres with superparamagnetic properties. Since polymerization occurs within mini-emulsion droplets, the spherical shape of the monomer droplets is retained in the final polymer particles, serving as a soft template. As polymerization progresses, the crosslinked PDVB structure separates from the DVB monomer, forming rough surface features on the microspheres. The walnut-like morphology arises from a combination of high polymerization degree, phase separation, and contraction of the crosslinked network. Two key factors influencing the final structure are radiation time and monomer composition; insufficient irradiation results in collapsed hollow microspheres, while limited phase separation in the St/PSt system prevents the development of walnut-like surface roughness. Magnetic characterization confirms that the microspheres exhibited superparamagnetic properties and high magnetic induction at ambient temperature, making them potentially useful for bio-applications, including targeted drug delivery, magnetic resonance imaging (MRI) contrast agents, hyperthermia treatment for cancer, and biomolecule separation.

## Figures and Tables

**Table 1:** Experimental conditions for preparing magnetite/polymer microspheres *.

Sample	Absorbed Dose (KGy)	Styrene (g)	Divinyl Benzene (DVB) (g)
S-1	18	0	3
S-2	48	0	3
S-3	66	0	3
S-4	72	0	3
S-5	72	3	0

* Other experimental conditions were the same as those in the typical experiment.

## Data Availability

The original contributions presented in this study are included in the article.

## Supplementary Materials

The following supporting information can be downloaded at: https://www.mdpi.com/article/10.3390/polym17131876/s1, Figure S1. FTIR spectrum of oleic acid modified MNPs. Figure S2. TGA curve of oleic acid modified MNPs.
