# Supplementary material for: Design and Synthesis of Fe3O4-Loaded Polymer Microspheres with Controlled Morphology: Section II Fabrication of Walnut-like Superparamagnetic Polymer Microspheres
Source: Polymers (Basel). 2025 Jul 5;17(13):1876. doi: 10.3390/polym17131876 (PMC12251648; doi:10.3390/polym17131876)
Supplement: Supplementary file 1 [file polymers-17-01876-s001.zip › polymers-3696585-supplementary.pdf]

Design and Synthesis of Fe_3O_4-Loaded Polymer Microspheres with Controlled Morphology: Section II Fabrication of Walnut-like Superparamagnetic Polymer Microspheres

Florence Acha, Talya Scheff, Nathalia DiazArmas and Jinde Zhang *

Department of Plastics Engineering, University of Massachusetts Lowell, Lowell, MA 01854, USA; florence_acha@student.uml.edu (F.A.); talya_scheff@student.uml.edu (T.S.); nathalia_diazarmas@student.uml.edu (N.D.)

* Correspondence: jinde_zhang@uml.edu; Tel.: +1-978-934-4896

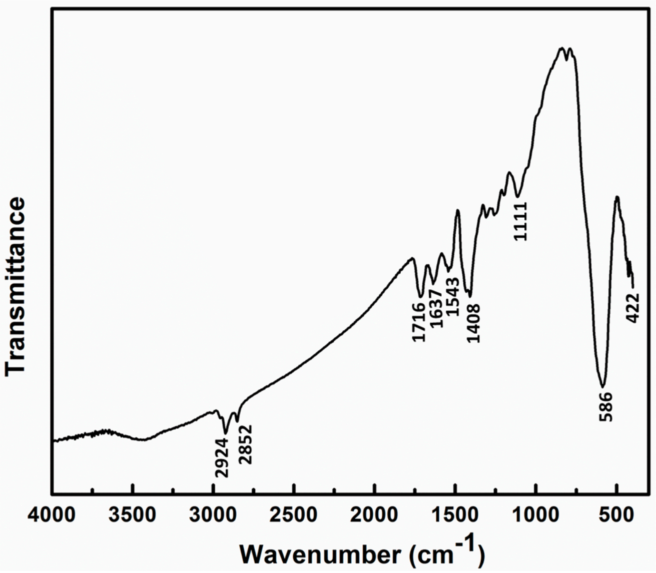

Figure S1: FTIR spectrum of oleic acid modified MNPs.

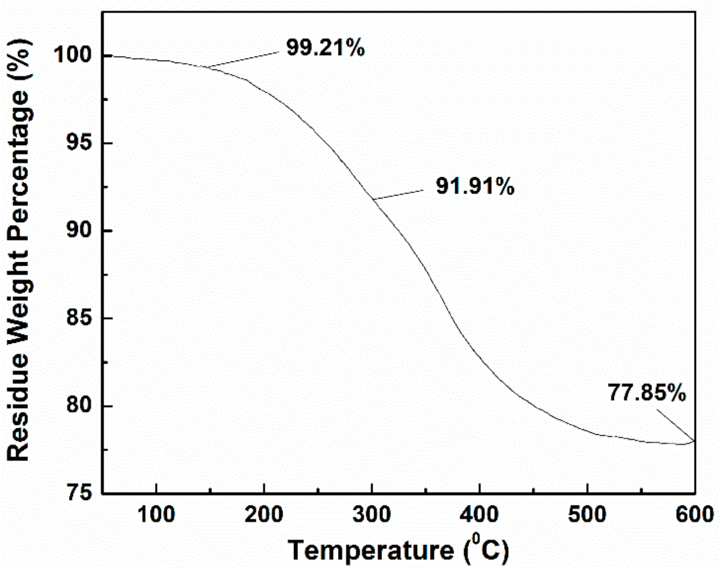

Figure S2. TGA curve of oleic acid modified MNPs
